# Comparative gel‐based proteomic analysis of chemically crosslinked complexes in dystrophic skeletal muscle

**DOI:** 10.1002/elps.201800028

**Published:** 2018-06-01

**Authors:** Sandra Murphy, Margit Zweyer, Rustam R. Mundegar, Dieter Swandulla, Kay Ohlendieck

**Affiliations:** ^1^ Department of Biology Maynooth University National University of Ireland Maynooth Co. Kildare Ireland; ^2^ Institute of Physiology II University of Bonn Bonn Germany

**Keywords:** Caveolin, Crosslinking mass spectrometry, Dystrophinopathy, Myoferlin, Trifunctional enzyme

## Abstract

Duchenne muscular dystrophy is a highly progressive muscle wasting disease with a complex pathophysiology that is based on primary abnormalities in the dystrophin gene. In order to study potential changes in the oligomerization of high‐molecular‐mass protein complexes in dystrophic skeletal muscle, chemical crosslinking was combined with mass spectrometric analysis. The biochemical stabilization of protein interactions was carried out with the homo‐bifunctional and amine‐reactive agent bis[sulfosuccinimidyl]suberate, followed by protein shift analysis in one‐dimensional gels. The proteomic approach identified 11 and 15 protein species in wild type versus dystrophic microsomal fractions, respectively, as well as eight common proteins, with an electrophoretic mobility shift to very high molecular mass following chemical crosslinking. In dystrophin‐deficient preparations, several protein species with an increased tendency of oligomerisation were identified as components of the sarcolemma and its associated intra‐ and extracellular structures, as well as mitochondria. This included the sarcolemmal proteins myoferlin and caveolin, the cytoskeletal components vimentin and tubulin, extracellular collagen alpha‐1(XII) and the mitochondrial trifunctional enzyme and oxoglutarate dehydrogenase. These changes are probably related to structural and metabolic adaptations, especially cellular repair processes, which agrees with the increased oligomerisation of myosin‐3, myosin‐9 and actin, and their role in cellular regeneration and structural adjustments in dystrophinopathy.

**Abbreviation**
BS^3^: bis(sulfosuccinimidyl)suberate

## 1 Introduction

The precise assembly and maintenance of supramolecular protein complexes and dynamic interaction patterns between protein subunits within membrane structures play key roles in many basic developmental, metabolic and physiological processes. It is therefore not surprising that abnormal quaternary protein structures and impaired protein complex formation are involved in the molecular mechanisms that underlie various disease mechanisms. A representative example of a collapsed protein complex being the disease initiator of a multifaceted pathological process is the loss of the dystrophin‐glycoprotein complex in the highly progressive neuromuscular disorder Duchenne muscular dystrophy 1, 2, 3. The primary abnormality in the *Dmd* gene causes the almost complete loss of its full‐length protein product, the Dp427 isoform of the membrane cytoskeletal protein dystrophin 4, and concomitant reduction in all members of the core dystrophin‐associated glycoprotein complex 5.

In healthy muscle tissues, the dystrophin complex provides a stabilizing linkage between the basal lamina and the membrane cytoskeleton 6, as well as forms a dynamic scaffold for signalling mechanisms at the fibre periphery 7. A large number of cell biological, biochemical, physiological, transcriptomic and proteomic studies have established intricate molecular and cellular changes downstream of the disintegration of the dystrophin‐glycoprotein complex. An impaired dystrophin assembly clearly destabilizes the sarcolemma and renders contractile fibres more susceptible to membrane micro‐rupturing, Ca^2+^‐induced necrosis, impaired excitation‐contraction coupling, sterile inflammation and reactive myofibrosis 8, 9, 10. The complexity of the dystrophin network and its involvement in the initiation of progressive fibre wasting in X‐linked muscular dystrophy therefore warrants a detailed assessment of protein complex alterations in normal versus dystrophic muscle tissue.

Adaptive or pathobiochemical changes in macromolecular oligomerization can be studied by a variety of highly advanced bioanalytical techniques, including X‐ray crystallography, nuclear magnetic resonance spectroscopy, Förster resonance energy transfer, and high‐resolution cryo‐electron microscopy, as well as yeast two‐hybrid screening, differential co‐immuno precipitation analysis, native two‐dimensional gel electrophoresis and gel filtration analysis. Another frequently used approach to profile the structure of protein complexes and interactions within supramolecular assemblies is chemical crosslinking (XL) 11, 12, 13. In the post‐genomic era, XL analysis and mass spectrometry (MS) have been successfully combined and are now routinely used as an integrated technology for structural analyses 14. The various methodological variations of crosslinking/mass spectrometry (XL/MS) and the establishment of bioinformatics programs for determining the production of stabile peptide crosslinks following protein digestion have been extensively reviewed 15, 16, 17. In contrast to the routine cataloguing of crosslinked peptides by XL/MS and auxiliary software screening for the estimation of intra‐ versus inter‐molecular protein interactions, we have used here a modified XL/MS approach for comparative purposes.

The analytical workflow consisted of (i) the subcellular enrichment of microsomal membranes from normal versus dystrophic muscle, (ii) the stabilization of protein‐protein interactions via the 11.4‐Å crosslinker bis[sulfosuccinimidyl]suberate (BS^3^), (iii) the comparative one‐dimensional gel electrophoretic separation of crosslinked molecules for protein shift analysis, (iv) in‐gel digestion to generate distinct peptide populations, and (v) the peptide mass spectrometric identification of stabilized proteins that exist in apparent supramolecular complexes. Thus, in contrast to standardized XL/MS approaches that directly determine crosslinked peptide structures and involve extensive bioinformatics, this investigation has determined the effect of muscular dystrophy on large protein complexes by comparative gel‐shift XL analysis. The altered electrophoretic mobility of protein species in wild type versus mutant muscle membranes in the presence of an XL agent was investigated in relation to specific molecular mass ranges in polyacrylamide gels. This bioanalytical approach builds on previous studies that have successfully combined comparative XL analysis with gel electrophoresis and immunoblotting 18, 19, 20.

The employed XL BS^3^ is an established analytical agent in protein biochemistry 21. BS^3^ is water‐soluble, homo‐bifunctional, non‐cleavable and amine‐reactive making this XL molecule especially suitable to stabilize complex protein structures 22, 23. The XL‐protein conjugation reaction can be carried out under experimental circumstances that are relatively close to physiological conditions. The comparative proteomic study identified a considerable number of protein species in the dystrophic microsomal fraction that exhibit a greatly reduced electrophoretic mobility following chemical crosslinking. Various proteins with an enhanced tendency for oligomerization were shown to be located in the sarcolemma, cytoskeletal networks, the extracellular matrix and mitochondria, and are mostly involved in membrane repair, fibre regeneration and oxidative metabolism. These findings suggest that distinct adaptations in critical protein‐protein interaction patterns exist that may counter‐act progressive fibre wasting in muscular dystrophy.

## 2 Materials and methods

### 2.1 Materials

For the gel‐based proteomic profiling of chemically cross‐linked protein species in wild type versus dystrophic *mdx‐4cv* skeletal muscle, analytical grade reagents and materials were purchased from GE Healthcare (Little Chalfont, Buckinghamshire, UK), Sigma Chemical Company (Dorset, UK), Bio‐Rad Laboratories (Hemel‐Hempstead, Hertfordshire, UK) and National Diagnostics (Atlanta, GA, USA). Protease inhibitor cocktails were obtained from Roche Diagnostics (Mannheim, Germany). The chemical cross‐linker BS³ and C18 spin columns were supplied by Thermo Fisher Scientific (Dublin, Ireland). Proteolytic digestion was carried out with sequencing grade modified trypsin from Promega (Madison, WI, USA). Biobasic C18 Picofrit columns were from Dionex (Sunnyvale, CA, USA).

### 2.2 Preparation of crude microsomes from skeletal muscle

The preparation of crude microsomes was performed as described previously 24. Skeletal muscle specimens were obtained from the Animal Facility of the University of Bonn and transported as quick‐frozen tissue samples to Maynooth University on dry ice in accordance with the Department of Agriculture animal by‐product register number 2016/16 (Department of Biology, Maynooth University). Briefly, 0.4 g of tissue from combined muscles of the hind leg from 5‐month old wild‐type C57Bl/6 (n = 4) and age‐matched dystrophic *mdx‐4cv* mice (n = 4) was finely chopped and homogenised in ten volumes of homogenisation buffer (20 mM sodium pyrophosphate, 20 mM sodium phosphate, 1 mM MgCl₂, 0.303 M sucrose, 0.5 mM EDTA, pH 7.0; supplemented with a protease inhibitor cocktail 25), using a hand‐held IKA T10 Basic Homogeniser (IKA Labortechnik, Staufen, Germany).

Following homogenisation, crude homogenates were incubated at 4°C for 2 h and then centrifuged at 14 000 × *g* for 20 min at 4°C using an Eppendorf 5417 R centrifuge (Eppendorf, Hamburg, Germany). The protein‐containing supernatant was carefully isolated and transferred to 4.9 ml Optiseal tubes. Samples were then centrifuged at 100 000 × *g* for 1 h at 4°C using an Optima L‐100 XP ultracentrifuge from Beckman Coulter (Fullerton, CA, USA) 25. Following ultracentrifugation, the resulting supernatant was removed and the pellet‐containing crude microsomes were re‐suspended in an appropriate volume of homogenisation buffer and stored at ‐20°C until required for chemical crosslinking.

### 2.3 Chemical crosslinking analysis of wild‐type versus *mdx‐4cv* microsomes

Protein concentrations were determined by the method of Bradford 26 and all samples were diluted to a concentration of 2 mg protein/ml with 50 mM HEPES, pH 8.0. The water‐soluble cross‐linker bis(sulfosuccinimidyl)suberate (BS³) was dissolved at a concentration of 1mg/ml in 50 mM citrate buffer, pH 5.0 18. Initial optimisation studies were used to evaluate various concentrations of BS³, including 0.1, 0.5, 1, 2, 5, 7.5, 10, 25, 50, 75, 100 and 150 μg cross‐linker per mg protein (not shown). For the main analysis described in this report, 10 μg BS³ per mg protein was selected. Following the addition of BS³, samples were incubated at 25°C for 30 min. The crosslinking reaction was quenched by the addition of 50 μL 1 M ammonium acetate per ml reaction mixture 19. An equal volume of reducing sample buffer was added and the samples were subsequently heated at 50°C for 10 min. Crosslinked samples were then electrophoresed alongside their non‐crosslinked counterparts on 1D SDS‐PAGE gels 20. For silver stain analysis 27, total loading was 10 μg protein per lane, while 30 μg protein was loaded per lane for Coomassie staining and subsequent in‐gel digestion.

### 2.4 In‐gel digestion of muscle proteins

In‐gel digestion for mass spectrometric analysis was performed as per the method of Shevchenko et al. 28. Protein lanes were cut into five separate segments A‐E and were processed separately. Individual Coomassie Blue‐stained gel zones were de‐stained by the addition of 100 μL of 100 mM ammonium bicarbonate:neat acetonitrile (1:1) solution, and incubated at 37°C for 30 min with gentle agitation. The solution was removed and 500 μL neat acetonitrile was added to each gel zone and incubated at room temperature for 10 min with gentle agitation. The solution was removed and gel pieces then underwent in‐gel trypsin digestion. Depending on the size of the gel piece, 50–100 μL of re‐suspended trypsin was added to each gel zone and incubated at 4°C for 30 min to allow slow diffusion of trypsin into the gel. A further 20 μL of trypsin buffer was added, and gel zones were incubated for 90 min at 4°C. 40 μL of a 50 mM ammonium bicarbonate solution was added and left to incubate overnight at 37°C. 100 μL extraction buffer [5% formic acid/neat acetonitrile (1:2)] was added to gel pieces and incubated at 37°C for 15 min with agitation. The supernatant, containing peptides, was transferred to fresh tubes, and dried down by vacuum centrifugation. Dried peptides were re‐suspended in 0.5% TFA/5% ACN, purified by C18 spin columns and dried by vacuum centrifugation 24. Dried peptides were stored at ‐80°C prior to mass spectrometric analysis.

### 2.5 Liquid chromatography mass spectrometry

Dried peptides were re‐suspended in loading buffer consisting of 2% ACN and 0.05% TFA in LC‐MS grade water. The LC‐MS/MS analysis of peptides was performed using an Ultimate 3000 NanoLC system (Dionex Corporation, Sunnyvale, CA, USA) coupled to a Q‐Exactive mass spectrometer (Thermo Fisher Scientific). 300 ng of each digested sample was loaded by an autosampler onto a C18 trap column (C18 PepMap, 300 μm id × 5 mm, 5 μm particle size, 100 Å pore size; Thermo Fisher Scientific). The trap column was switched on‐line with an analytical Biobasic C18 Picofrit column (C18 PepMap, 75 μm id × 50 cm, 2 μm particle size, 100 Å pore size; Dionex). Peptides were eluted using a 65 min method over the following binary gradient [solvent A: (2% (v/v) ACN and 0.1% (v/v)) formic acid in LC‐MS grade water and solvent B: 80% (v/v) ACN and 0.1% (v/v) formic acid in LC‐MS grade water]: 3% solvent B for 5 min, 3–10% solvent B for 5 min, 10–40% solvent B for 30 min, 40–90% solvent B for 5 min, 90% solvent B for 5 min and 3% solvent B for 10 min 29. The column flow rate was set to 0.3 μL/min. Data were acquired with Xcalibur software (Thermo Fisher Scientific). The Q‐Exactive mass spectrometer was externally calibrated and operated in positive, data‐dependent mode. A full survey MS scan was performed in the 300–1700 m/z range with a resolution of 140 000 (m/z 200) and a lock mass of 445.12003. Collision‐induced dissociation (CID) fragmentation was carried out with the fifteen most intense ions per scan and at 17 500 resolution. A dynamic exclusion window was applied within 30 s. An isolation window of 2 m/z and one microscan were used to collect suitable tandem mass spectra.

### 2.6 Proteomic identification of muscle‐associated protein species

Qualitative analysis of the mass spectrometry raw files was performed using Proteome Discoverer 1.4 against Sequest HT (SEQUEST HT algorithm, licence Thermo Scientific, registered trademark University of Washington, USA) using the UniProtKB/Swiss‐Prot database, with 25 041 sequences for mouse (*mus musculus*). The following search parameters were used for protein identification: (i) peptide mass tolerance set to 10 ppm, (ii) MS/MS mass tolerance set to 0.02 Da, (iii) an allowance of up to two missed cleavages, (iv) carbamidomethylation set as a fixed modification and (v) methionine oxidation set as a variable modification. Peptides were filtered using a minimum XCorr score of 1.5 for 1, 2.0 for 2, 2.25 for 3 and 2.5 for four charge states, with peptide probability set to high confidence 30. For the identification of common versus unique protein species, proteins were only considered to be unique if they were detected in all four replicates of one condition and in none of the four replicates in the other condition. Proteins which are considered common or shared between control and crosslinked samples are those which are detected in all four of the control replicates and all four of the crosslinked replicates for a given gel piece.

To generate Venn diagrams for the illustration of common versus unique proteins in the four analyzed cohorts (wild‐type control, wild‐type crosslinked, *mdx‐4cv* control and *mdx‐4cv* crosslinked proteins), Venny 2.1 software (http://bioinfogp.cnb.csic.es/tools/venny/) was used. Potential protein interactions amongst unique crosslinked proteins in wild‐type and *mdx‐4cv* samples were analyzed by version 10.5 of the STRING database (http://string-db.org/) for medium confidence (0.4) interactions with experimental evidence. The STRING analysis programme clusters proteins based on known and predicted protein interactions that include direct physical and indirect functional protein associations 31.

## 3 Results

### 3.1 Chemical crosslinking analysis of microsomal membranes

**Figure 1:**
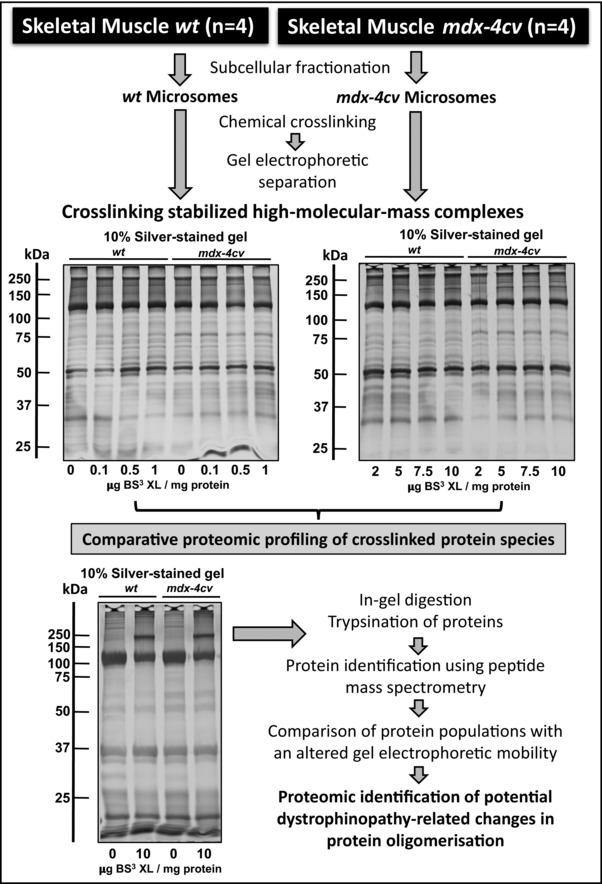
Gel‐based chemical crosslinking and mass spectrometric analysis of microsomes from dystrophic skeletal muscle. Shown is a flowchart that outlines the analytical approach to estimate increased tendencies of protein oligomerisation in wild‐type (*wt*) versus dystrophic *mdx‐4cv* muscle preparations. In the upper panel, the silver‐stained SDS‐PAGE gels represent the analysis of microsomes that were incubated with 0, 0.1, 0.5 and 1, as well as 2, 5, 7.5 and 10 μg cross‐linker bis(sulfosuccinimidyl)suberate (BS³) per mg protein in *wt* versus *mdx‐4cv* preparations. Lanes 1 to 4 in the silver‐stained gel in the lower panel represent non‐treated *wt* muscle versus 10 μg BS³/mg protein‐incubated *wt* muscle versus non‐treated *mdx‐4cv* muscle versus 10 μg BS³/mg protein‐incubated *mdx‐4cv* muscle preparations, respectively. Molecular mass standards (in kDa) are indicated on the left of gel images.

In order to investigate potential changes in the oligomeric status of muscle‐associated proteins due to deficiency in the membrane cytoskeletal component dystrophin, chemical crosslinking was combined with gel electrophoretic shift analysis and mass spectrometry. The increased tendency of protein‐protein interactions within high‐molecular‐mass complexes was determined with the amine‐reactive and homo‐bifunctional crosslinker BS^3^. The flowchart in Fig. 1 outlines the bioanalytical approach used in this study and shows representative silver‐stained gel images of protein shifting in 1D SDS‐PAGE gels following incubation with the water‐soluble and non‐cleavable agent BS^3^. In microsomal preparations from both wild‐type and dystrophic skeletal muscle, increasing XL concentration (0.1–1 and 2–10 μg BS^3^ per mg protein) triggered distinct changes in the protein band pattern. Such alterations in the protein banding pattern included the appearance of intense bands at approximately 150 kDa and above 250 kDa in crosslinked samples, particularly at concentrations of 5, 7.5 and 10 μg BS^3^ per mg protein (Supporting Information Figs. 1 and 2). Alterations in the protein composition of different gel regions was carried out by LC‐MS/MS analysis, comparing non‐treated wild‐type muscle versus XL‐incubated wild‐type muscle versus non‐treated dystrophic muscle versus XL‐incubated dystrophic muscle preparations.

### 3.2 Mass spectrometric identification of proteins with altered electrophoretic mobility

**Figure 2:**
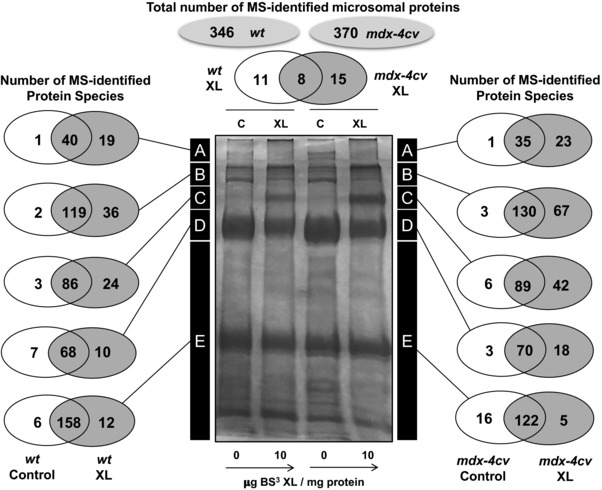
Mass spectrometric identification of crosslinked muscle proteins with an altered electrophoretic mobility. Shown is a Coomassie‐stained SDS‐PAGE gel with chemically crosslinked microsomes from wild‐type (*wt*) versus dystrophic *mdx‐4cv* skeletal muscle. Lanes 1 to 4 are non‐treated *wt* muscle versus 10 μg BS³/mg protein‐incubated *wt* muscle versus non‐treated *mdx‐4cv* muscle versus 10 μg BS³/mg protein‐incubated *mdx‐4cv* muscle preparations, respectively. The number of MS‐identified proteins in the high to low molecular mass zones A‐E of the analysed gel lanes is illustrated by Venn diagrams.

The comparative proteomic approach presented here focused on the systematic analysis of the XL‐induced reduction in gel electrophoretic mobility of muscle proteins in normal versus dystrophic skeletal muscle. The shift to gel regions of higher molecular mass was combined with sensitive MS analysis and resulted in the unequivocal identification of 346 and 370 protein species in wild‐type versus dystrophic preparations, respectively. Figure 2 summarizes the number of MS‐identified proteins in the high to low molecular mass zones of the analysed gels, denoted as A to E. The results suggest that a considerable number of proteins exhibit tight protein‐protein interaction patterns that can be stabilized by incubation with an XL chemical. Although the majority of microsomal proteins did not undergo a drastic alteration in electrophoretic mobility following XL treatment, distinct cohorts of protein species exhibited a BS^3^‐dependent shift to higher molecular masses. The focus of our proteomic investigation was on the class of very high‐molecular‐mass complexes, which represent various supramolecular assemblies of the muscle membrane system and its associated intra‐ and extracellular structures.

The comparative analysis of the protein species with a drastically reduced gel electrophoretic mobility following XL treatment identified 11 and 15 protein species in wild‐type versus dystrophic microsomal fractions, respectively, as well as 8 common proteins in zone A of the Coomassie Blue‐stained gel shown in Fig. 2. The mass spectrometric identification of altered proteins in normal versus *mdx4cv* preparations is listed in Tables 1 and 2, respectively. A list of commonly detected proteins is presented in Supporting Information Table 1, and peptide information for Tables 1 and 2 can be found in Supporting Information Table 2. In addition, files with the mass spectrometric identification of all proteins in zones A‐E in control versus crosslinked *wt* samples and control versus crosslinked *mdx‐4cv* samples (Fig. 2) are available as Supporting Information Tables 3 and 4.

**Table 1:** Mass spectrometric identification of proteins with a reduced gel electrophoretic mobility following chemical crosslinking of the microsomal fraction from wild‐type mouse skeletal muscle

Accession number	Protein name	Percentage coverage	Unique peptides
P11531	Dystrophin Dp427	5.98	17
Q3V1D3	AMP deaminase 1	19.06	9
P11499	Heat shock protein HSP 90‐beta	8.98	6
O08638	Myosin‐11	5.83	6
Q9Z1E4	Glycogen [starch] synthase, muscle	8.27	5
Q91YQ5	Dolichyl‐diphosphooligosaccharide–protein glycosyltransferase subunit 1	9.05	4
Q9R0Y5	Adenylate kinase isoenzyme 1	23.2	3
P97447	Four and a half LIM domains protein 1	11.07	3
P43274	Histone H1.4	15.53	2
P04247	Myoglobin	21.43	2
O88492	Perilipin‐4	12.83	2

**Table 2:** Mass spectrometric identification of proteins with a reduced gel electrophoretic mobility following chemical crosslinking of the microsomal fraction from dystrophic *mdx‐4cv* mouse skeletal muscle

Accession number	Protein name	Percentage coverage	Unique peptides
Q8BMS1	Trifunctional enzyme subunit alpha, mitochondrial	19.92	13
Q8VDD5	Myosin‐9	7.4	13
Q7TQ48	Sarcalumenin	17.25	12
Q60597	2‐oxoglutarate dehydrogenase, mitochondrial	14.71	12
Q60847	Collagen alpha‐1(XII) chain	5.1	7
Q8BFR5	Elongation factor Tu, mitochondrial	16.81	6
Q99JY0	Trifunctional enzyme subunit beta, mitochondrial	12.84	6
Q9D2G2	Dihydrolipoyllysine‐residue succinyltransferase component of 2‐oxoglutarate dehydrogenase complex, mitochondrial	8.37	4
Q7TSH2	Phosphorylase b kinase regulatory subunit beta	5.25	4
P13541	Myosin‐3	19.38	3
P49817	Caveolin‐1	19.05	3
Q69ZN7	Myoferlin	1.92	3
P60710	Actin, cytoplasmic 1	26.4	2
P99024	Tubulin beta‐5 chain	15.32	2
P20152	Vimentin	9.23	2

**Figure 3:**
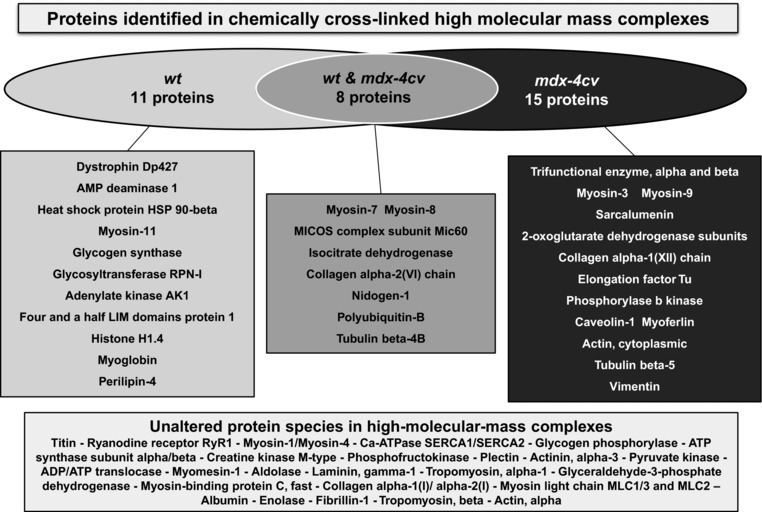
Overview of muscle protein species with a drastically reduced gel electrophoretic mobility following chemical crosslinking analysis. Shown are comparative listings of proteins with a tendency to increased oligomerisation in normal versus dystrophic microsomes. Details of the MS‐based identification of altered proteins in control versus *mdx‐4cv* preparations are listed in Tables 1 and 2, respectively.

The exclusive presence of the full‐length Dp427 isoform of dystrophin in the fraction from normal muscle confirmed the mutant status of the analysed specimens. Other members of the core dystrophin‐glycoprotein complex were not identified in this gel zone representing extremely high‐molecular‐mass assemblies. This is probably due to the relatively low abundance, small size and hydrophobicity of dystrophin‐associated proteins 2, 6. Additional proteins with a tendency to form large complexes included various metabolic enzymes, structural elements, molecular chaperones and transporters. A large number of very large muscle proteins, such as titin and the ryanodine receptor calcium release channel, as well as proteins that naturally form large molecular clusters including calcium pumps, contractile proteins and glycolytic enzymes, are listed in the lower part of Fig. 3 and were shown to be present in the upper region of SDS‐PAGE gels irrespective of the presence of XL agents.

### 3.3 Mass spectrometric identification of altered proteins in muscular dystrophy

In microsomal preparations from *mdx‐4cv* skeletal muscle, 15 protein species with a distinct gel electrophoretic mobility shift were identified by MS analysis (Table 2). The individual proteins are located in diverse subcellular regions such as the sarcolemma, the sarcoplasmic reticulum, the nucleus, the extracellular matrix, the cytoskeleton, the cytosol and mitochondria. Identified proteins fall into the main functional categories of ion homeostasis, cellular regulation, structural maintenance, muscle contraction, metabolism and cellular repair. An increased tendency of oligomerisation was shown to occur in myosin heavy chain isoforms myosin‐3 and myosin‐9, the luminal Ca^2+^‐binding protein sarcalumenin of the sarcoplasmic reticulum, the elongation factor Tu of the translational apparatus of mitochondria, the trifunctional enzyme of the mitochondrial fatty acid beta‐oxidation pathway, tubulin and vimentin, the extracellular matrix collagen isoform alpha‐1(XII), components of the 2‐oxoglutarate dehydrogenase complex, phosphorylase b kinase, cytoplasmic actin, the surface membrane protein caveolin‐1 and the sarcolemma repair protein myoferlin (Fig. 3).

### 3.4 Bioinformatic analysis of proteomic changes in dystrophic muscle

**Figure 4:**
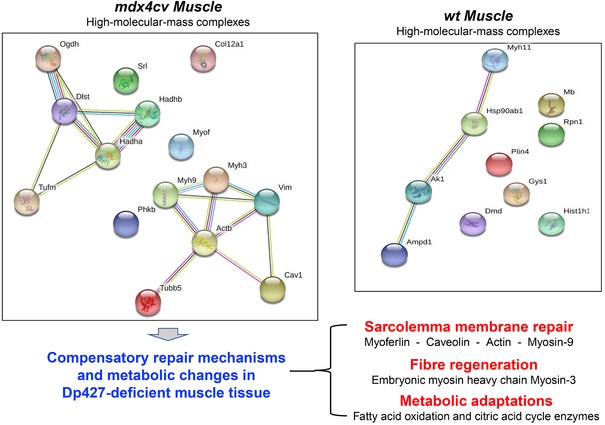
Bioinformatic analysis of proteomic changes in the microsomal fraction from dystrophic muscle. Shown are the findings from the bioinformatic analysis of protein interactions using *STRING* 31 software programs.

The bioinformatic analysis using the *STRING* program 31 was used to determine whether any of the identified muscle‐associated proteins with an increased tendency to form oligomeric structures may belong to common complexes, pathways or cellular processes. Figure 4 shows the findings from the protein interaction analysis and illustrates that in normal muscle a minor hub appears to exist between the heat shock protein Hsp90, myosin‐11, adenylate kinase isoform AK1 and AMP deaminase 1. The protein interaction analysis of highly oligomerised protein species in dystrophin‐deficient microsomes suggests a cluster of up‐regulated myosin heavy chains and the cytoskeletal proteins vimentin and tubulin. Potential pathways include the membrane repair and wound healing complex consisting of myoferlin, caveolin, actin and myosin, which is probably involved in compensatory fibre regeneration processes to counteract progressive muscle wasting. The increased complex formation of mitochondrial enzymes involved in fatty acid oxidation and the citric acid cycle indicates metabolic adaptations in dystrophic fibres.

## 4 Discussion

The almost complete loss of the membrane cytoskeletal protein dystrophin renders skeletal muscle fibres more susceptible to necrosis. The disintegration of the dystrophin‐associated glycoprotein complex causes sarcolemmal instability, which in turn results in abnormal Ca^2+^‐fluxes and high levels of proteolytic degradation 10. Progressive muscle wasting, sterile inflammation and reactive myofibrosis are characteristic features of X‐linked muscular dystrophy. These complex pathophysiological changes are reflected by distinct proteome‐wide alterations, encompassing a large spectrum of proteins involved in ion handling, signal transduction, the excitation‐contraction‐relaxation cycle, the innate immune response, bioenergetic pathways, metabolic regulation and the cellular stress response 24, 25, 30, 32, 33, 34, 35, 36. The findings of the combined XL and MS study presented here suggest that numerous muscle‐associated proteins with an altered abundance also exhibit a changed oligomerisation pattern. A variety of physiological regulators, structural components and repair molecules showed a drastically increased tendency to form protein‐protein interactions in response to dystrophin deficiency.

In contrast to conventional gel electrophoretic shift analyses, which routinely employ immunoblotting for the detection of altered mobility patterns 18, 19, 20, the method outlined in this report has used the more reliable MS‐based identification of changes in muscle proteins. Often monoclonal or monospecific antibodies to monomeric subunits do not recognize their respective antigen(s) in XL‐stabilized protein complexes, making the determination of the range of protein oligomerisation difficult. In addition, hypothesis‐driven immunoblotting surveys following XL incubation with a select number of antibodies are inherently biased. As shown in this technology‐driven study, the number of altered proteins with a change in their oligomeric status is considerable. In comparison to the relatively limited scope of immunoblot analyses, the efficient and unequivocal identification of protein changes by the highly sensitive LC‐MS/MS method is more comprehensive and can be combined with systems bioinformatics.

The increased tendency of oligomerisation of different components of the mitochondrial trifunctional enzyme and the oxoglutarate dehydrogenase complex is an interesting finding in relation to potential metabolic changes and bioenergetic adaptations in muscular dystrophy. The trifunctional enzyme complex of the inner mitochondrial membrane system is an important component that mediates fatty acid utilization and altered enzymatic functionality has severe pathophysiological consequences 37. A previous survey of crude microsomal membranes by MS‐based proteomics has revealed elevated levels of this metabolic enzyme in dystrophin‐deficient skeletal muscle tissue 38. The 2‐oxoglutarate dehydrogenase complex is a multi‐enzyme assembly of the mitochondrial matrix that catalyses the overall conversion of 2‐oxoglutarate to succinyl‐CoA and CO_2_ as a crucial part of the citric acid cycle in skeletal muscle 39. Oxoglutarate dehydrogenase also undergoes stress‐mediated changes and may therefore play a role in the cellular stress response 40. The analysis of muscle cell cultures from Duchenne patients and dystrophin‐deficient animal models has revealed structural changes in mitochondria and altered activity levels of antioxidant and mitochondrial enzymes 41, 42. Based on these observations, an attractive and alternative pathomechanism to the main calcium hypothesis of dystrophinopathy has been suggested, i.e. systemic mitochondrial impairments play a key role in X‐linked muscular dystrophy 43. Thus, the increased complex formation of the mitochondrial trifunctional enzyme and the oxoglutarate dehydrogenase may represent an adaptive process to counter‐act impaired lipid metabolism and insufficient ATP production in dystrophic fibres. Mitochondrial dysfunction appears to contribute to the pathological muscle wasting syndrome by reducing availability of ATP needed for essential calcium regulation and fibre regeneration 44. In addition, various XL‐stabilised protein species were shown to be present in both normal and dystrophic muscle fractions, including components of the sarcomere such as myosin heavy chain isoforms 7 and 8, the protein degradation element polyubiquitin‐B, metabolic enzymes and the cytoskeletal tubulin subunit beta‐4B, as well as the extracellular matrix components nidogen‐1 and collagen alpha‐2(VI).

Importantly, the MS‐based identification of muscular dystrophy‐related changes in protein oligomerisation agrees with the identification of altered protein species in previous comparative proteomic surveys of dystrophic skeletal muscles 24, 30, 35, 38. The XL‐focused investigation described here suggests that the loss of the membrane cytoskeletal protein dystrophin and resulting sarcolemmal instability is probably compensated by the up‐regulation of the cytoskeletal proteins vimentin and tubulin in the fibre interior 30. Although high concentration levels of vimentin are usually only transiently observed during the maturation of myotubes 45, this cytoskeletal protein can act synergistically to desmin 46. The compensatory increase in vimentin, which appears to occur in all dystrophic muscle subtypes 47, and the elevated levels of vimentin protein interaction levels, as indicated by the findings of the XL analysis described in this report, suggests a vimentin‐associated support of the structural backbone of intermediate filaments. This probably restores, at least partially, the load‐bearing function of contractile fibres in the absence of the cytoskeletal dystrophin lattice.

The changes in myoferlin are most likely related to the initiation of cellular repair processes. Myoferlin and dysferlin are involved in important regulatory processes, including transverse tubule formation and Ca^2+^‐handling in skeletal muscles 48. In conjunction with annexins and actin, the repair proteins myoferlin and dysferlin play an important role in skeletal muscle membrane fusion and restoration mechanisms in muscular dystrophy 49. The efficient resealing of the dystrophin‐deficient sarcolemma by vesicular patching presents a key protective response in muscular dystrophy. This process is probably supported by the increased oligomerisation of various isoforms of myosin and actin, as shown here by XL analysis. The above listed proteins are majorly involved in cellular regeneration and structural adaptations in dystrophinopathy. The increased oligomerisation of caveolin‐1 would agree with its elevated concentration in *mdx* muscle and increased numbers of vesicular invaginations of the sarcolemma, called caveolae, in dystrophic muscles 50. Caveolae structures are involved in signalling processes and endocytic trafficking, and changes in caveolin proteins appear to be related to regenerative processes 10, 51.

In conclusion, the combination of chemical crosslinking, electrophoretic gel‐shift analysis and mass spectrometry has been successfully applied to compare the tendency of protein oligomerisation in normal versus dystrophic skeletal muscle tissue. A variety of muscle‐associated protein species were shown to exhibit an elevated level of cluster formation. This included proteins involved in oxidative metabolism, ion homeostasis, membrane repair and fibre regeneration. The majority of these alterations in protein interaction patterns are probably in response to the loss of sarcolemmal integrity and represent compensatory mechanisms to rescue the dystrophic phenotype. In the future, the systematic establishment of these types of proteome‐wide changes might be exploitable to establish novel biomarker candidates for the improved diagnosis, prognosis and/or therapy‐monitoring in the field of muscular dystrophy.

*The authors have declared no conflict of interest*.

## Supporting information

Supporting Material

Supporting Material

Supporting Material

Supporting Material

Supporting Material

Supporting Material
