# Supplementary material for: Comparative gel‐based proteomic analysis of chemically crosslinked complexes in dystrophic skeletal muscle
Source: Electrophoresis. 2018 Jun 1;39(14):1735–44. doi: 10.1002/elps.201800028 (PMC6099379; doi:10.1002/elps.201800028)
Supplement: Supplementary file 1 — Supporting Material [file ELPS-39-1735-s001.pptx]

## Slide 1
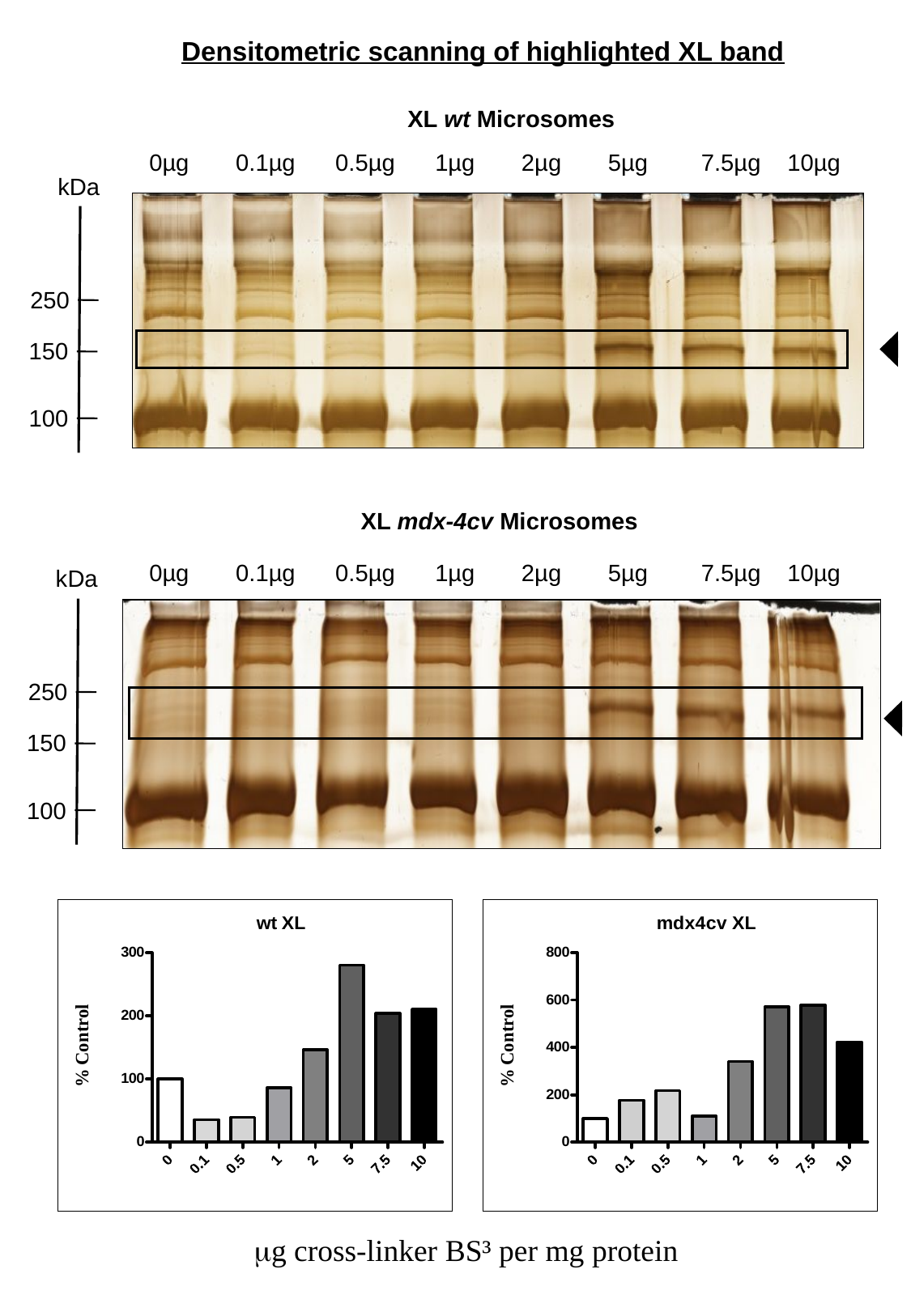

Densitometric scanning of highlighted XL band
XL wt Microsomes
0µg 0.1µg 0.5µg 1µg 2µg 5µg 7.5µg 10µg
kDa
250
150
100
XL mdx-4cv Microsomes
0µg 0.1µg 0.5µg 1µg 2µg 5µg 7.5µg 10µg
kDa
250
150
100
μg cross-linker BS³ per mg protein
