# Supplementary material for: Comparative gel‐based proteomic analysis of chemically crosslinked complexes in dystrophic skeletal muscle
Source: Electrophoresis. 2018 Jun 1;39(14):1735–44. doi: 10.1002/elps.201800028 (PMC6099379; doi:10.1002/elps.201800028)
Supplement: Supplementary file 2 — Supporting Material [file ELPS-39-1735-s002.pptx]

## Slide 1
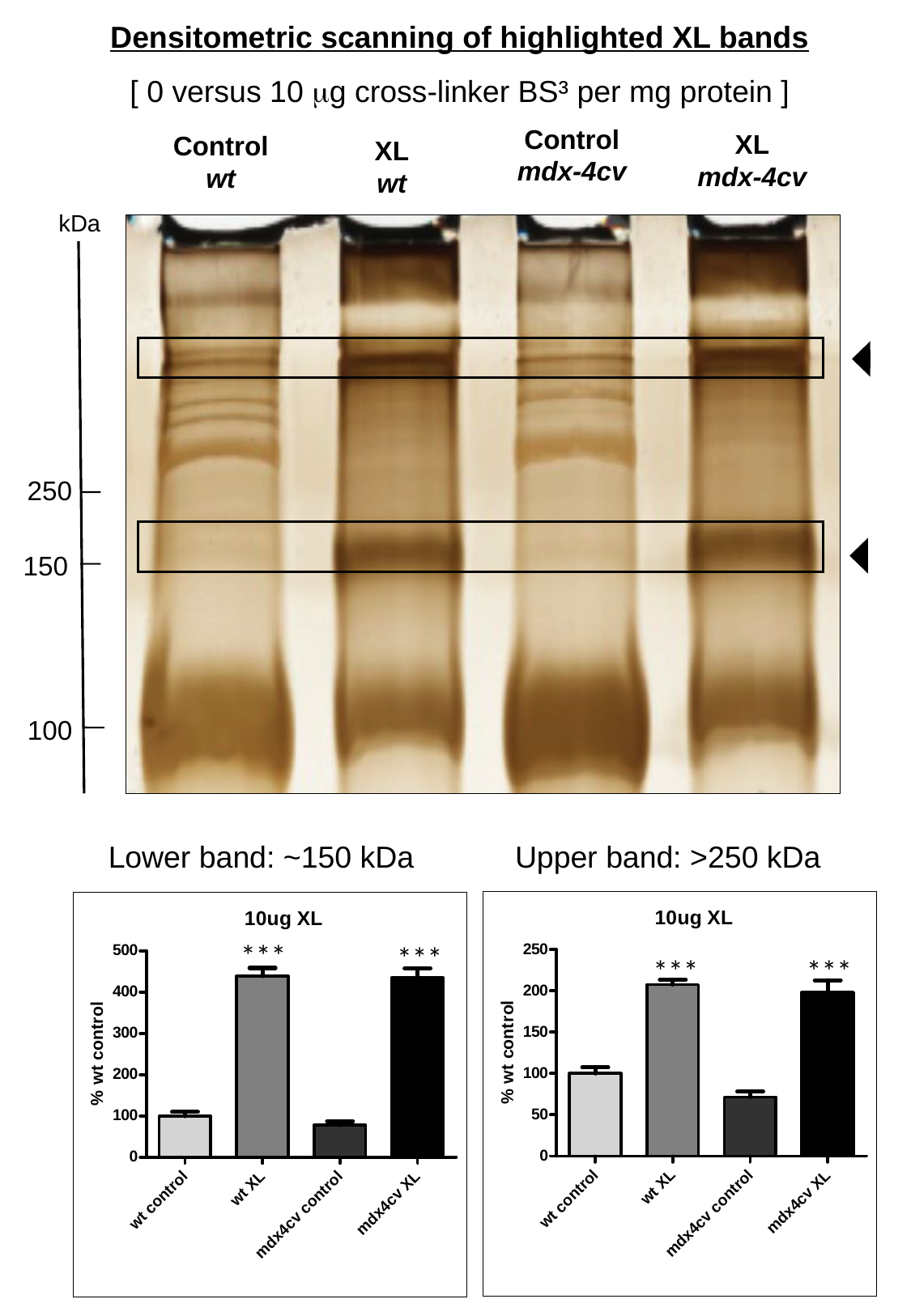

Densitometric scanning of highlighted XL bands
[ 0 versus 10 μg cross-linker BS³ per mg protein ]
Control
mdx-4cv
XL
mdx-4cv
Control
wt
XL
wt
kDa
250
150
100
Lower band: ~150 kDa
Upper band: >250 kDa
***
***
***
***
