# Supplementary material for: Comparative gel‐based proteomic analysis of chemically crosslinked complexes in dystrophic skeletal muscle
Source: Electrophoresis. 2018 Jun 1;39(14):1735–44. doi: 10.1002/elps.201800028 (PMC6099379; doi:10.1002/elps.201800028)
Supplement: Supplementary file 3 — Supporting Material [file ELPS-39-1735-s003.docx]

Table S1: **Mass spectrometric identification of proteins with a reduced gel electrophoretic mobility following chemical crosslinking of the microsomal fraction from both wild type and *mdx-4cv* mouse skeletal muscle**

| **Accession No.** | **Protein Name** | **% Coverage wt** | **Unique peptides wt** | **% Coverage mdx-4cv** | **Unique peptides mdx-4cv** |
| --- | --- | --- | --- | --- | --- |
| Q91Z83 | Myosin-7 | 32.87 | 25 | 25.68 | 17 |
| Q8CAQ8 | MICOS complex subunit Mic60 | 14.75 | 8 | 24.4 | 13 |
| Q9D6R2 | Isocitrate dehydrogenase [NAD] subunit alpha, mitochondrial | 21.58 | 6 | 13.11 | 4 |
| P13542 | Myosin-8 | 45.07 | 6 | 38.26 | 4 |
| Q02788 | Collagen alpha-2(VI) chain | 5.13 | 5 | 8.03 | 7 |
| P10493 | Nidogen-1 | 3.05 | 3 | 1.77 | 2 |
| P0CG49 | Polyubiquitin-B | 32.79 | 2 | 32.79 | 2 |
| P68372 | Tubulin beta-4B chain | 17.3 | 2 | 15.28 | 2 |
